# On the development of extragonadal and gonadal human germ cells

**DOI:** 10.1242/bio.013847

**Published:** 2016-02-01

**Authors:** A. Marijne Heeren, Nannan He, Aline F. de Souza, Angelique Goercharn-Ramlal, Liesbeth van Iperen, Matthias S. Roost, Maria M. Gomes Fernandes, Lucette A. J. van der Westerlaken, Susana M. Chuva de Sousa Lopes

**Affiliations:** 1 Department of Anatomy and Embryology, Leiden University Medical Center, Einthovenweg 20, 2333 ZC Leiden, The Netherlands; 2 Dept. of Veterinary Medicine, Faculty Animal Sciences and Food Engineering, University of São Paulo, Av. Duque de Caxias Norte 225, 13630-000 SP Pirassununga, Brazil; 3 Dept. of Surgery, Faculty Veterinary Medicine and Animal Sciences, University of São Paulo, Av. Prof. Dr. Orlando Marques Paiva 87, 05508-270 SP São Paulo, Brazil; 4 Department of Gynaecology, Leiden University Medical Center, Albinusdreef 2, 2333 ZA Leiden, The Netherlands; 5 Department for Reproductive Medicine, Ghent University Hospital, De Pintelaan 185, 9000 Ghent, Belgium

**Keywords:** Human, Fetal, Adrenals, Ovaries, Germ cells, Meiosis, Development, Ectopic

## Abstract

Human germ cells originate in an extragonadal location and have to migrate to colonize the gonadal primordia at around seven weeks of gestation (W7, or five weeks post conception). Many germ cells are lost along the way and should enter apoptosis, but some escape and can give rise to extragonadal germ cell tumors. Due to the common somatic origin of gonads and adrenal cortex, we investigated whether ectopic germ cells were present in the human adrenals. Germ cells expressing DDX4 and/or POU5F1 were present in male and female human adrenals in the first and second trimester. However, in contrast to what has been described in mice, where ‘adrenal’ and ‘ovarian’ germ cells seem to enter meiosis in synchrony, we were unable to observe meiotic entry in human ‘adrenal’ germ cells until W22. By contrast, ‘ovarian’ germ cells at W22 showed a pronounced asynchronous meiotic entry. Interestingly, we observed that immature POU5F1+ germ cells in both first and second trimester ovaries still expressed the neural crest marker TUBB3, reminiscent of their migratory phase. Our findings highlight species-specific differences in early gametogenesis between mice and humans. We report the presence of a population of ectopic germ cells in the human adrenals during development.

## Abstract (precis)

**Summary:** The presence and developmental trajectory of ectopic human germ cells in the adrenals and gonads during the first and second trimesters is investigated by comparing the expression dynamics of early, late and meiotic germ cell markers.

## INTRODUCTION

In humans, primordial germ cells (PGCs) originate outside the gonadal primordia, in the posterior part of the yolk sac, close to the allantois and hindgut wall and undergo a phase of proliferation and migration towards the gonadal ridge (Byskov, 1986; Mollgard et al., 2010). Normally, these PGCs reach gonadal primordia around week 7 of gestation (W7, or week 5 post conception) to become enclosed in either seminiferous tubules or ovarian cords, respectively in male and female embryos (Heeren et al., 2015).

However, some PGCs in humans may stop migrating along the way to the gonads (Mamsen et al., 2012) or become lodged in extragonadal organs. The most obvious ectopic organ to lodge PGCs would be the adrenal glands (or adrenals). This is because the somatic gonad and adrenal cortex, both steroid-producing organs, have a common somatic origin and both organs are colonized by migratory neural crest cells (Keegan and Hammer, 2002; Mollgard et al., 2010; Morohashi, 1997). In mice and bovine, ectopic germ cells have been described in the adrenal glands (Upadhyay and Zamboni, 1982; Wrobel and Suss, 1999; Zamboni and Upadhyay, 1983). Ectopic germ cells present along the migratory pathway are most of the times eliminated by apoptosis (Runyan et al., 2008; Stallock et al., 2003), however, when lodged in the adrenal glands some of these ectopic germ cells survive and are able to undergo meiosis to become oocytes in both females and males (Upadhyay and Zamboni, 1982; Zamboni and Upadhyay, 1983). This suggests that the adrenal glands may provide a microenvironment that induces (or allows) germ cells to undergo a female sex differentiation pathway. Interestingly, these adrenal ‘oocytes’ seem to develop synchronous with gonadal ‘oocytes’ regarding growth, meiotic entry and they even develop a zona pellucida (Zamboni and Upadhyay, 1983), in agreement with the current view of a default female pathway in the urogenital region, initiated by exposure to retinoic acid (RA) and blocked in the male gonad by local degradation of RA or related metabolites (Bowles et al., 2006; Koubova et al., 2006; Kumar et al., 2011).

In different animal models, PGCs follow different ‘routes’ to reach the gonads, including the gut and abdominal mesentery in mice (Starz-Gaiano and Lehmann, 2001) or the vasculature in chicken (De Melo Bernardo et al., 2012). Interestingly, it has been suggested that in humans, PGCs may migrate along the peripheral nervous system (Mamsen et al., 2012; Mollgard et al., 2010). Sympathetic nerve fibers were also found in the adrenal glands and indeed human ectopic PGCs could enter the adrenal glands via these fibers (Mamsen et al., 2012).

Here, we have investigated the presence of ectopic human germ cells in the adrenals during human development (from W8.4 until W22) and investigated how these ectopic germ cells developed by comparing the dynamics of expression of early, late and meiotic germ cell markers between the adrenal and the gonadal germ cells. ‘Adrenal’ germ cells seem to upregulate the late marker DDX4, but we were unable to observe ‘adrenal’ germ cells entering meiosis until W22. However, we show that meiotic entry in human female gonads is an asynchronous process that is still taking place after W22. We discuss possible ways of how the human germ cells reach the adrenals and on the fate of those ‘adrenal’ germ cells.

## RESULTS

### Germ cells were present in several ectopic locations in female mice embryos

**Fig. 1.**
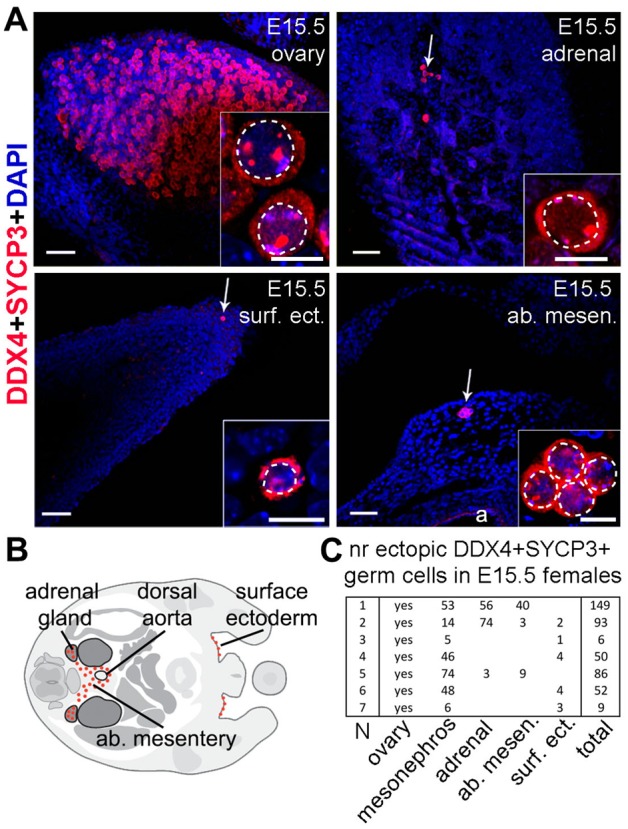
**Ectopic germ cells in mice female embryos at E15.5.** (A) Transverse sections of E15.5 female embryos showing DDX4+/SYCP3+ germ cells (white arrows) at several anatomical locations (ovary, adrenal glands, surface ectoderm and abdominal mesentery). Magnified views show germ cells in more detail and dashed line depicts border between nucleus and cytoplasm. Abbreviations: a, aorta; surf. ect., surface ectoderm; ab. mesen., abdominal mesentery. Scale bars are 50 µm, and in the magnified inserts 5 µm. (B) Schematic of locations where ectopic germ cells were observed. (C) Table of the number of double DDX4+/SYCP3+ germ cells per ectopic location in each female E15.5 embryo analyzed (*n*=7).

First, we investigated the presence of germ cells at ectopic locations in mice at embryonic day (E)15.5 when basically all female gonadal germ cells have entered meiosis (Bullejos and Koopman, 2004). Using immunofluorescence for the late germ cell marker DDX4 (or VASA) and the meiotic marker SYCP3, we observed the presence of DDX4-positive and SYCP3-positive germ cells in the E15.5 females analyzed (*n*=7) not only in the ovaries, but we also observed some germ cells in the mesonephros, sometimes in the abdominal mesentery, peri-aortic region, adrenal glands and a few in the proximity of the surface ectoderm, close to the external genital region and base of tail (Fig. 1A-C).

### Germ cells were present in human fetal male and female adrenals

**Fig. 2.**
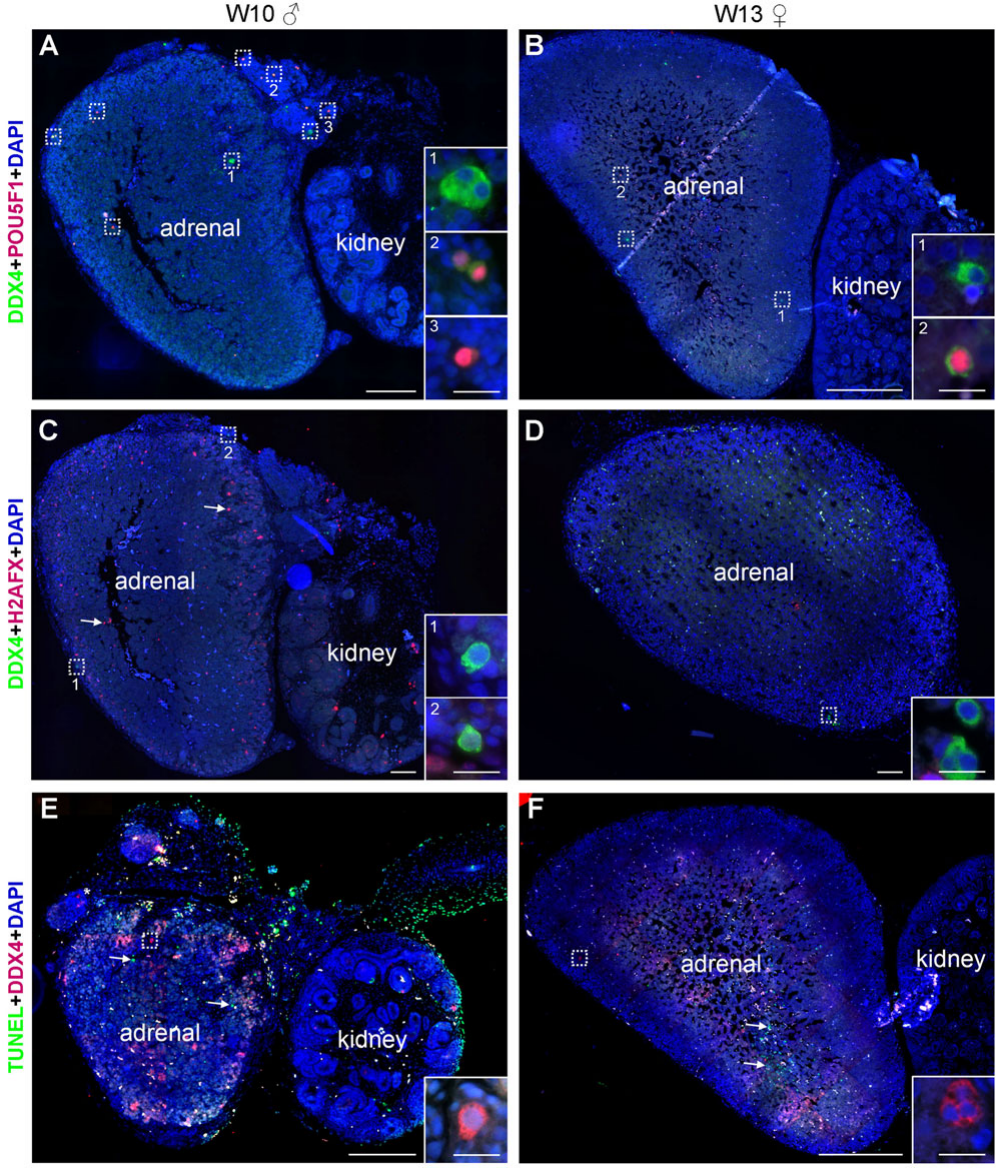
**Germ cells in first trimester human fetal adrenals.** Histological sections of human adrenals (and adjacent kidney) in first trimester male (W10) and female (W13). (A,B) Germ cells were identified by expression of POU5F1 (red) and/or DDX4 (green). (C,D) DDX4+ (green) germ cells do not express H2AFX (red), however, many H2AFX+ cells were present in the adrenals (white arrows). (E,F) DDX4+ (red) germ cells were not TUNEL-positive (green), however, many TUNEL+ cells were present in the adrenals (white arrows). Note the presence of autofluorescent red blood cells. Some germ cells are highlighted in dotted boxes. Magnified views of dotted boxes are shown as inserts and the insert number corresponds to its location. Scale bars are 200 µm in A,B,E,F, 100 µm in C,D and 20 µm in all inserts.

Next, we investigated the presence of germ cells in human fetal adrenal glands in females and in males (Table S1) by immunofluorescence for DDX4 and the early germ cell marker POU5F1 (or OCT4). We were able to detect single and double POU5F1/DDX4-positive germ cells, sometimes in small clusters, in all first trimester adrenal glands analyzed from W8.4-W13 (Fig. 2A,B; Fig. S1). We quantified consecutive sections of several adrenals (*n*=3 females and *n*=3 males) and the majority of the sections contained 1 to 10 germ cells (Table 1). Moreover, POU5F1/DDX4-positive germ cells were never detected in the kidneys, when those were present in the slides.

**Table 1.**
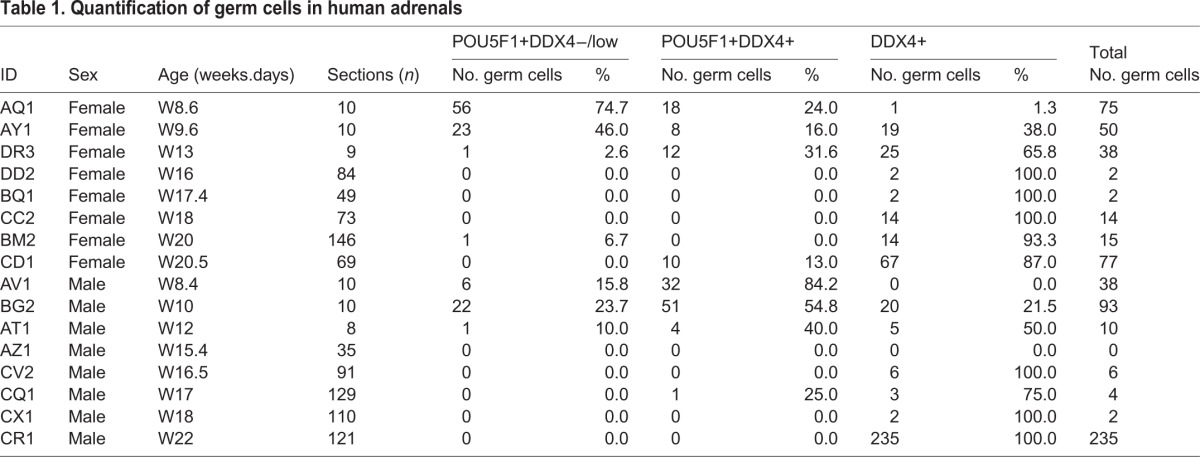
**Quantification of germ cells in human adrenals**

To understand whether these ‘adrenal’ germ cells were entering meiosis, we used immunofluorescence for DDX4 and either (phosphorylated) H2AFX, a marker associated with DNA double-strand breaks both during meiosis entry, but also apoptosis (Hunter et al., 2001; Ismail and Hendzel, 2008; Rogakou et al., 1999) or SYCP3. Both in female and male first trimester adrenals, DDX4+ germ cells were never positive for H2AFX (Fig. 2C,D; Fig. S1) or SYCP3 (Fig. S2). However, many H2AFX+ cells were observed in the adrenals and could correspond to adrenal cells undergoing apoptosis. To further investigate the fate of DDX4+ cells in the adrenals, we also performed TUNEL assay (Fig. 2E,F; Fig. S1), but were unable to observe ‘adrenal’ DDX4+ germ cells in apoptosis. We conclude that during first trimester, pre-meiotic germ cells are relatively abundant in the adrenals (Fig. 2).

**Fig. 3.**
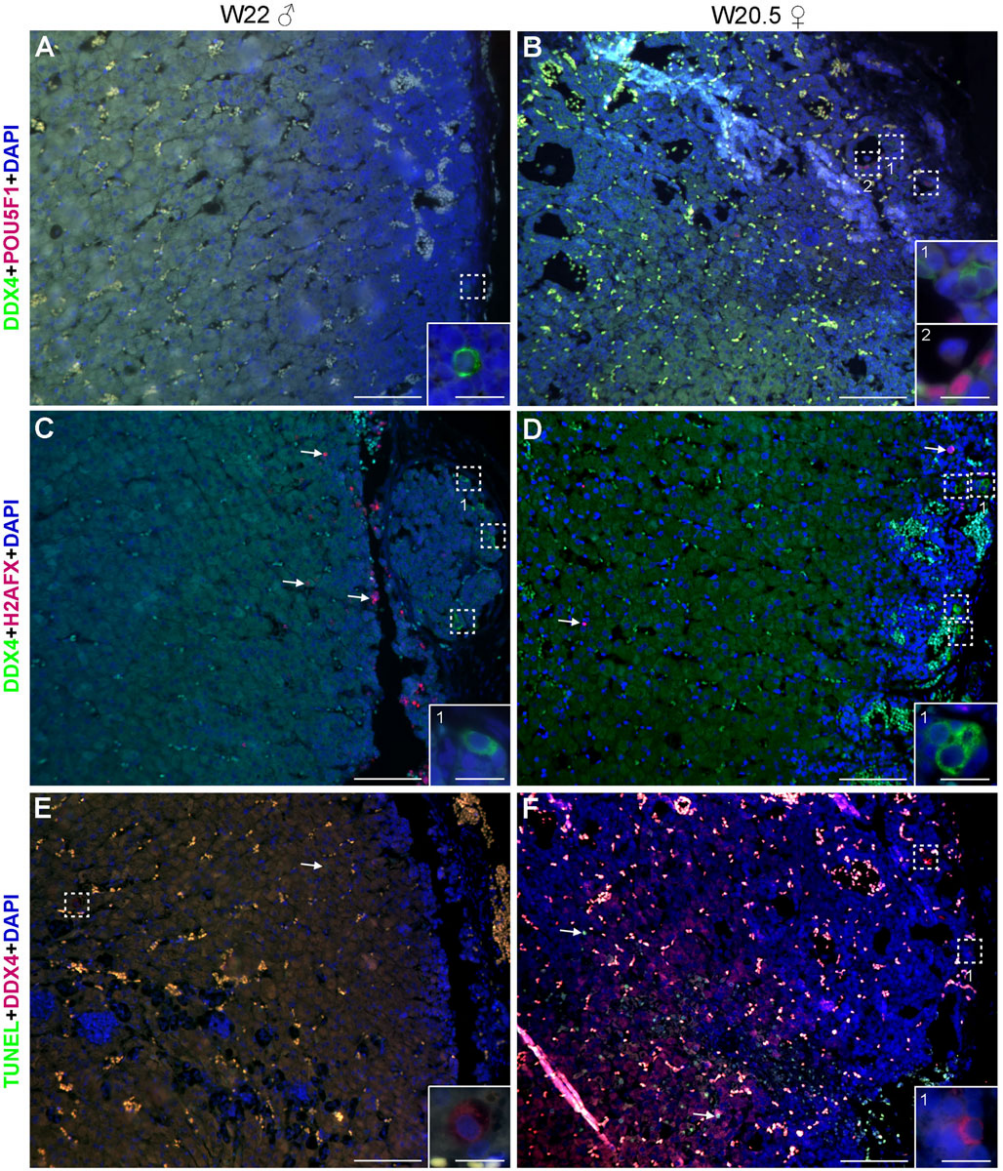
**Germ cells in second trimester human fetal adrenals.** Histological sections of human adrenals of second trimester male (W22) and female (W20.5). (A,B) Germ cells were identified by expression of POU5F1 (red) and/or DDX4 (green). (C,D) DDX4+ (green) germ cells do not express H2AFX (red), however, H2AFX+ cells were present in the adrenals (white arrows). (E,F) DDX4+ (red) germ cells were not TUNEL-positive (green), however TUNEL+ cells were present in the adrenals (white arrows). Note the presence of autofluorescent red blood cells. Some germ cells are highlighted in dotted boxes. Magnified views of dotted boxes are shown as inserts and the insert number corresponds to its location. Scale bars are 100 µm in A-F and 20 µm in all inserts.

We proceeded analyzing both female and male second trimester adrenals ranging from W14-W22, where we encountered germ cells at the periphery of the adrenal (Fig. 3A,B, Table 1; Fig. S3), but we were unable to detect H2AFX+ germ cells (Fig. 3C,D; Fig. S3) or SYCP3+ germ cells (Fig. S2). From the few germ cells encountered none were positive for TUNEL (Fig. 3E,F; Fig. S3). Our results in human are in contrast with results in mice showing that germ cells observed in the adrenals were able to survive and mature (Upadhyay and Zamboni, 1982; Zamboni and Upadhyay, 1983).

### Developmental dynamics of human germ cells in first and second trimester

**Fig. 4.**
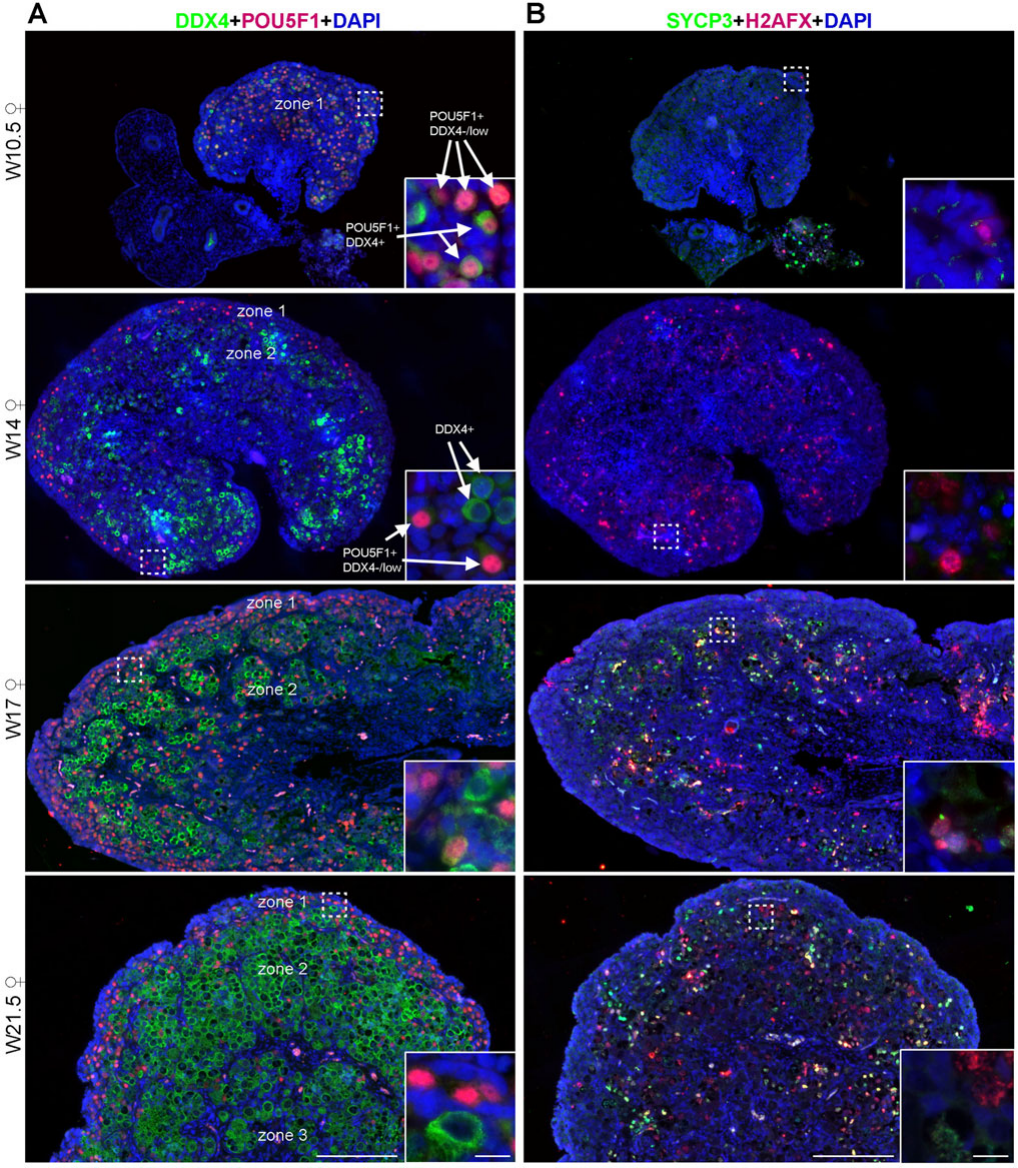
**Dynamic expression of germ cell markers in human fetal ovaries.** (A) Histological sections of human ovaries at W10.5, W14, W17 and W21.5, immunostained for the early germ cell marker (nuclear) POU5F1 (red) and late germ cell marker (cytoplasmic) DDX4 (green). In zone 1, most germ cells are POU5F1+DDX4−/low; in zone 2 and 3, most germ cells are DDX4+. Several germ cells in zone 3 have developed into primordial follicles. Inserts are magnifications of the dotted boxes. (B) Histological sections of human ovaries at W10.5, W14, W17 and W21.5, immunostained for the meiotic markers H2AFX (red) and SYCP3 (green). Inserts are magnifications of the dotted boxes. Note the presence of autofluorescent red blood cells. Scale bars are 200 µm and in the magnified inserts 20 µm.

As we were unable to observe meiotic entry in the human ‘adrenal’ germ cells until W22, we investigated the spatial dynamics of meiotic entry in human ovaries, when possible from the same individual from which we had analyzed the adrenals (Table S1). We discriminated firstly between POU5F1+ and DDX4+ germ cells (Fig. 4A) and, secondly, we immunostained consecutive sections for two independent meiotic markers, H2AFX and SYCP3 (Fig. 4B).

As previously observed by us and others (Anderson et al., 2007; Heeren et al., 2015), in first trimester ovaries most germ cells were POU5F1+ and expressed either low (or no) DDX4, consisting of a relatively homogeneous population of germ cells (zone 1) (Fig. 4A, Table 2; Fig. S4A). During second trimester, we observed a shift towards DDX4+ germ cells interiorly (zone 2), whereas a pronounced peripheral layer of POU5F1+ cells remained (Fig. 4A, Table 2; Fig. S4B). At W21.5, the majority of germ cells in the ovary were DDX4+POU5F1− and localized interiorly. In addition, some of these DDX4+ cells were developing into primordial follicles (zone 3) (Fig. 4A).

**Table 2.**
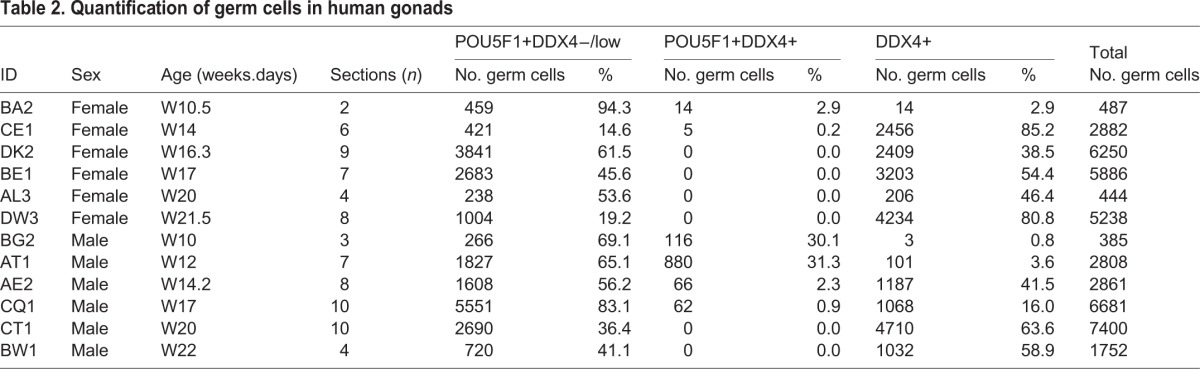
**Quantification of germ cells in human gonads**

**Fig. 5.**
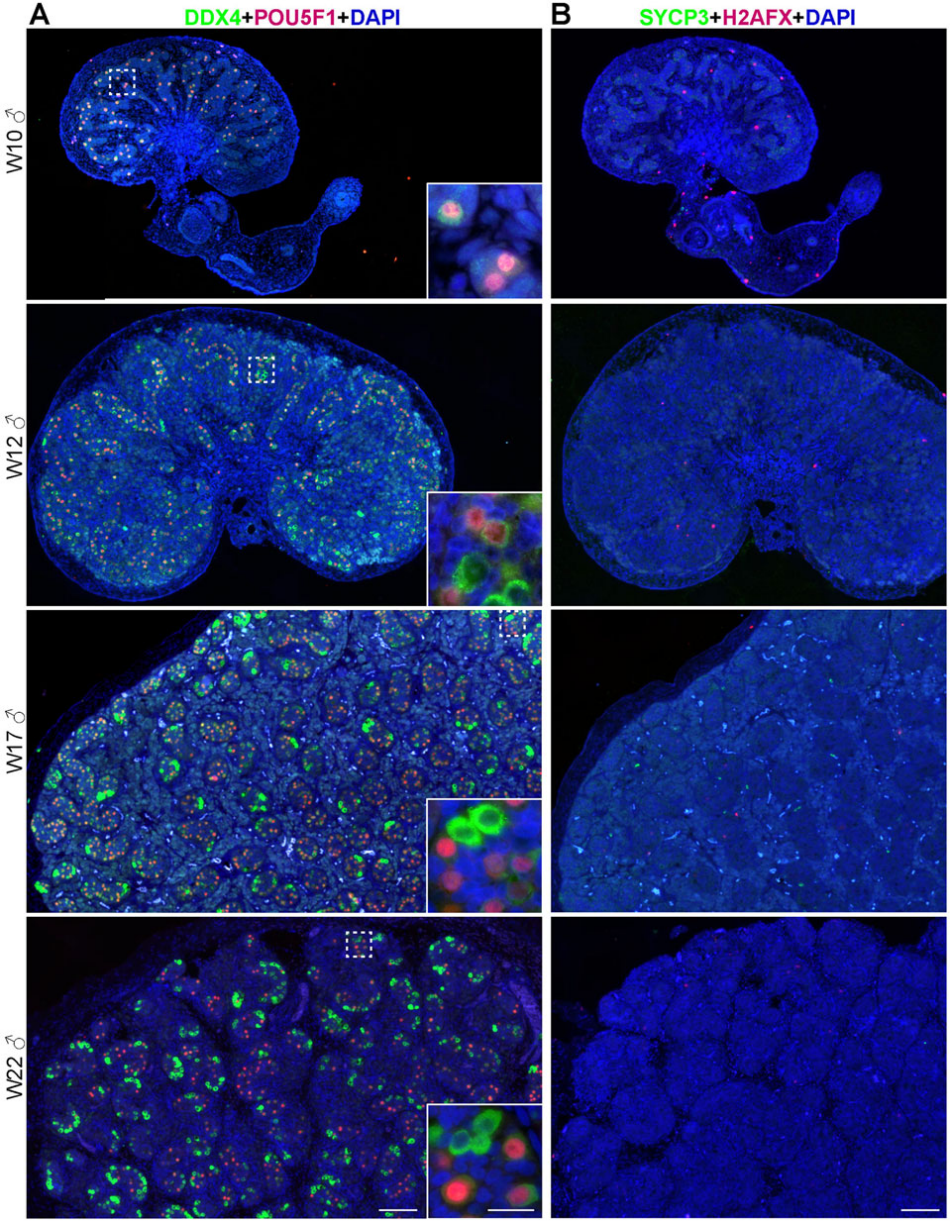
**Dynamic expression of germ cell markers in human fetal testes.** (A) Histological sections of human testes at W10, W12, W17 and W22, immunostained for the early germ cell marker (nuclear) POU5F1 (red) and late germ cell marker (cytoplasmic) DDX4 (green). Inserts are magnifications of the dotted boxes. (B) Histological sections of human ovaries at W10, W12, W17 and W22, immunostained for the meiotic markers H2AFX (red) and SYCP3 (green). Note the presence of autofluorescent red blood cells. Scale bars are 200 µm and in the magnified inserts 20 µm.

Germ cells in male gonads were also quantified and, as in females, a shift towards DDX4+POU5F1− germ cells was observed between the first and second trimesters (Fig. 5A, Table 2; Fig. S5). Until W22, male germ cells expressed neither H2AFX nor SYCP3 (Fig. 5B).

### Asynchronous meiotic entry in human ovarian germ cells

**Fig. 6.**
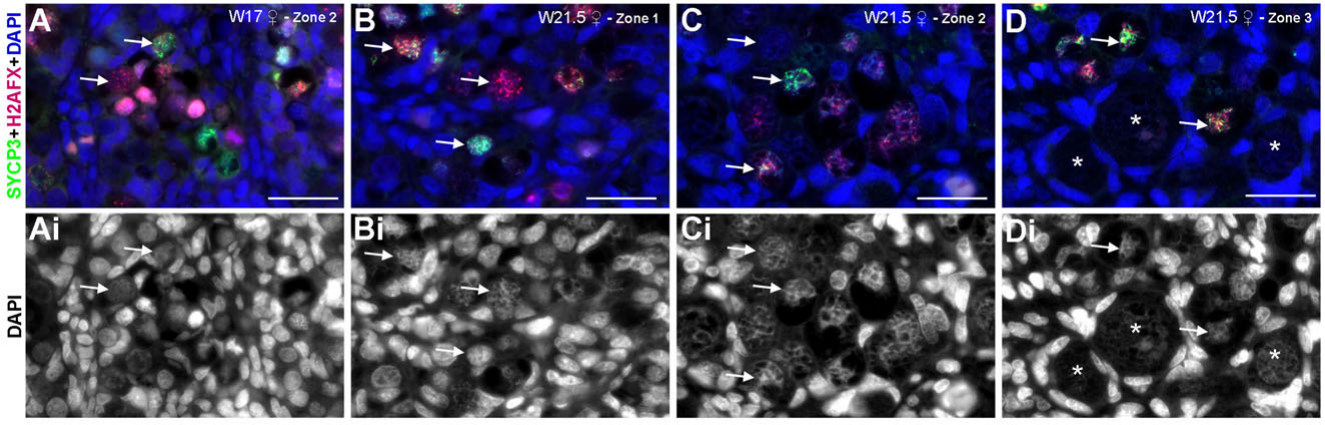
**Meiosis in second trimester human fetal ovaries.** (A) High magnifications of histological sections of a human ovary at W17 immunostained for the meiotic markers H2AFX (red) and SYCP3 (green) showing zone 2. (B-D) High magnifications of histological sections of a human ovary at W21.5, immunostained for the meiotic markers H2AFX (red) and SYCP3 (green) showing zone 1, zone 2 and zone 3. White arrows point to germ cells showing decondensed chromatin and various degrees of H2AFX and SYCP3 expression. Note that primordial follicles (indicated by white asterisks), characteristic of zone 3, were negative for both H2AFX and SYCP3. Ai,Bi,Ci,Di, DAPI channel only. Scale bars are 30 µm.

In human ovaries, H2AFX was upregulated in many germ cells at W14 (Fig. 4B). However, at W17, germ cells in zone 2 (where most DDX4+ germ cells were located) showed chromatin decondensation and interestingly either predominately H2AFX or SYCP3 filament-like expression (Fig. 4B, Fig. 6A,Ai), suggestive of meiotic entry. By contrast, cells in the peripheral area (zone 1), where most POU5F1+ germ cells located remained negative for both meiotic markers (Fig. 4B). Later, at W21.5, as zone 1 becomes thinner, more peripheral germ cells started showing chromatin decondensation and a salt-and-pepper pattern expression of H2AFX, SYCP3 or both (Fig. 4B; Fig. 6B,Bi,C,Ci). In primary follicles in zone 3, the oocytes expressed neither H2AFX nor SYCP3 (Fig. 6D,Di). However, it is clear that many germ cells in zone 1, most probably POU5F1+DDX4−/low germ cells, had not entered meiosis by W21.5. Comparing our data with that reported for spread human spermatocytes double-stained for H2AFX and SYCP3 (Roig et al., 2004), we conclude that H2AFX+SYCP3−/low germ cells were in (pre-)leptotene, germ cells containing patches of H2AFX and SYCP3 were in zygotene; SYCP3+H2AFX− germ cells were in pachytene and the SYCP3−H2AFX− oocytes in primordial follicles, as the synaptonemal complexes dissolve, have reached diplotene.

It has been reported that human germ cells show marked asynchrony regarding meiotic phases during fetal ovarian development (Kurilo, 1981) and this is in agreement with our data. However, even though we observed a maturation wave from peripheral POU5F1+ germ cells to the more inner-located primordial follicles, we have no evidence to support the idea that the different prophase meiotic phases occur in a wave-like manner in the ovary.

### Association between human germ cells and neural crest derivatives

To further understand the relationship between the ‘ovarian’ and ‘adrenal’ germ cells, we studied the presence of neural crest derivatives in both adrenals and ovaries between W9 and W22. Neural crest derivatives, marked by class III beta-tubulin (TUBB3), have been described to guide human PGCs towards the gonadal primordia (Mamsen et al., 2012; Mollgard et al., 2010) and perhaps inadvertently to other organs, including the adrenals.

**Fig. 7.**
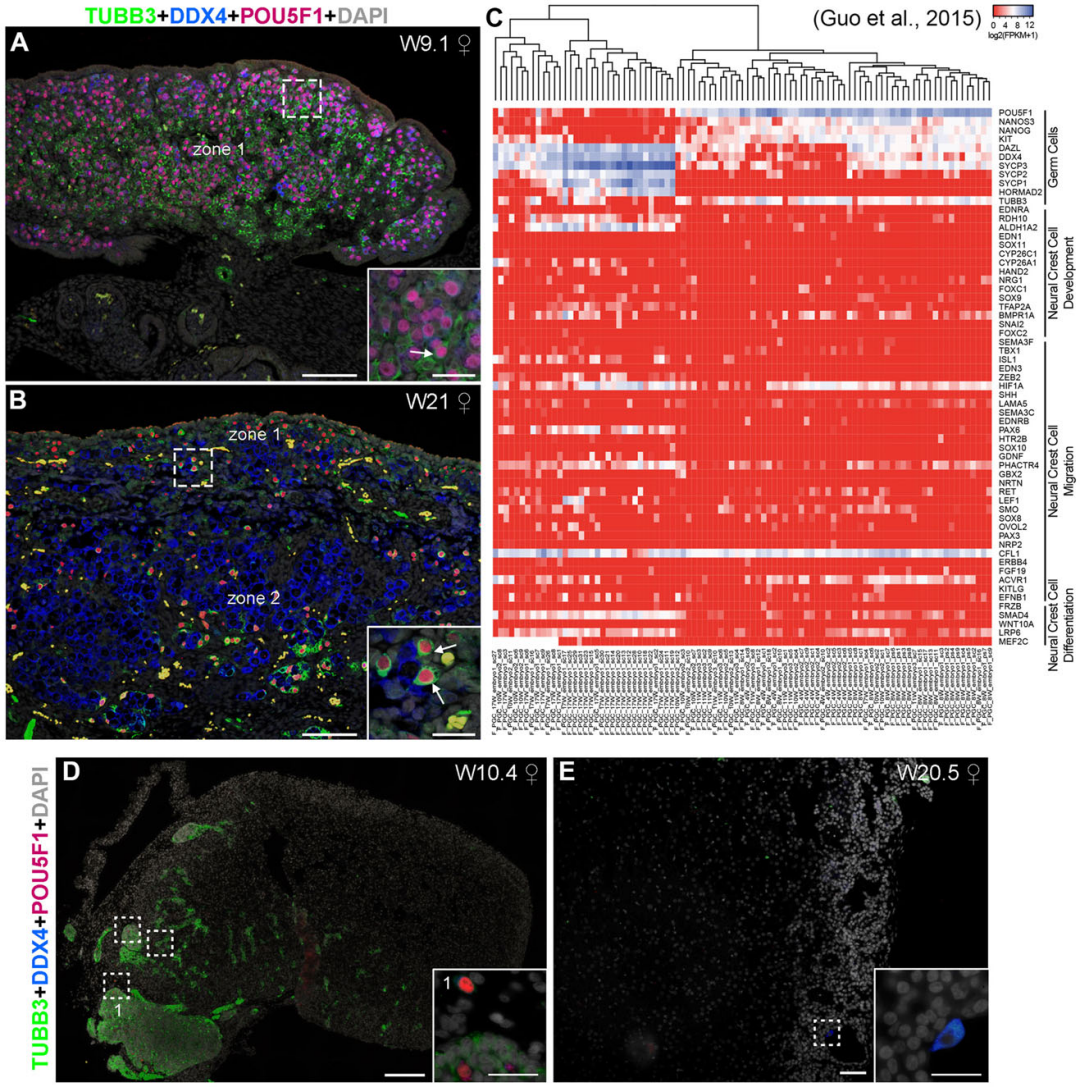
**Expression of TUBB3 in human fetal female ovaries and adrenals.** (A,B) Histological section of human ovaries at W9.1 (A) and W21 (B) immunostained for the early germ cell marker POU5F1 (red), late germ cell marker DDX4 (blue), TUBB3 (green) and DAPI (grey). Inserts are magnifications of the dotted boxes. White arrows point to germ cells expressing POU5F1 and TUBB3. Note the presence of autofluorescent red blood cells. (C) Heatmap depicting the expression profile in fragments per kilobase of transcript per million (FPKM) of selected genes including early, late and meiotic germ cell markers as well as neural crest development, migration and differentiation markers early in a data set consisting of single-cell transcriptional profile of human female germ cells of first and second trimester (Guo et al., 2015). (D,E) Histological section of human fetal female adrenals at W10.4 (D) and W20.5 (E) immunostained for the early germ cell marker POU5F1 (red), late germ cell marker DDX4 (blue), TUBB3 (green) and DAPI (grey). Inserts are magnifications of the dotted boxes. Scale bars are 100 µm in A,B,E; 200 µm in D and 20 µm in all inserts.

Surprisingly, we observed that most POU5F1+ germ cells in the ovaries from both first and second trimester were strongly positive for TUBB3 (Fig. 7A,B). However, most DDX4+ germ cells in the second trimester were TUBB3− (Fig. 7B). This was interesting because it suggested that early human germ cells could have a ‘neural crest’ related expression signature, reminiscent of their migratory phase. We are aware that TUBB3 is not an exclusive marker for migratory neural crest cells, but is a general marker for the neural lineage (Locher et al., 2014); and it is possible that this and many other genes are expressed in germ cells as a consequence of the global DNA demethylation that PGCs undergo during migration (Guo et al., 2015).

We investigated this further by interrogating an online available single cell transcriptomics dataset of human female germ cells from first and second trimester (Guo et al., 2015). Using early (POU5F1, NANOG, KIT) and late markers (DAZL, DDX4, SYCP3, SYCP2, SYCP1, HORMAD2) to differentiate between early and late human female germ cells, we confirmed that the majority of early POU5F1+DDX4−/low germ cells (in zone 1) expressed TUBB3, whereas late DDX4+ germ cells (zone 2) did not. However, except for TUBB3 and perhaps ACVR1 (or ALK2), a receptor associated with germ cell development in mice (Chuva de Sousa Lopes et al., 2004), we did not find a clear association between early germ cells and markers of neural crest cell development, migration or differentiation (Fig. 7C). Late DDX4+ germ cells were enriched for neural crest markers such as RDH10, ALDH1A2, PAX6, PHACTR4, SMAD4 and LRP6 (Fig. 7C).

We were able to detect many TUBB3+ nerve bundles penetrating the first trimester adrenals (to form the adrenal medulla) and there we could also observe POU5F1+ and/or DDX4+ germ cells (Fig. 7D), in agreement with others (Mamsen et al., 2012). The few peripheral DDX4+ cells encountered in the second trimester expressed no (or low) levels of TUBB3 (Fig. 7E), as in the ovary.

## DISCUSSION

### How do the human germ cells end up in the adrenals?

We observed that ‘ovarian’ and ‘adrenal’ POU5F1+ germ cells expressed TUBB3. As these germ cells are still phenotypically immature, expression of TUBB3 is a reminiscence of the migratory phase of germ cells and a common characteristic with neural crest derivatives (Mamsen et al., 2012; Mollgard et al., 2010). Whether germ cells only share this characteristic with neural crest cell derivatives or effectively depend on structural support provided by neural crest derivatives to colonize the gonadal ridge (or the adrenals) remains to be elucidated. We could envisage that neural crest cells and PGCs could use (partly) common migratory routes, share common markers, or respond to similar chemo-attractant cues to colonize both the adrenal and genital primordia.

An alternative explanation to the presence of germ cells in the adrenals is the common origin of the human gonadal primordia and adrenal primordia, both initially expressing high levels of DAX1 (also known as NR0B1) and SF1 (also known as NR5A1) (Hanley et al., 1999, 2001; Morohashi, 1997). The colonization by the PGCs, at least in rats, is shown to occur before the physical separation of gonadal and adrenal primordia (Hatano et al., 1996). It is feasible that in humans this also occurs and that some germ cells reach the adrenal primordia and remain there after the two organs separate.

### Do human ‘adrenal’ germ cells enter meiosis synchronously with ‘ovarian’ germ cells?

In mice, the meiotic wave (described from anterior to posterior) in females is of relatively short duration (from E11.5 to E12.5) and gametogenesis during mice embryonic development is further a rather synchronous process (Bowles and Koopman, 2007). By contrast, the process of gametogenesis from PGCs to primordial follicle in female human germ cells is strongly asynchronous and clearly spatially regulated (Anderson et al., 2007; Heeren et al., 2015). Here, we show that in humans the time window for the start of meiosis in females is less defined than in mouse and we did not observe robust meiotic entry (by SYCP3) at least until W17 in ovaries. However, even at W21.5 in the female gonads there was still a large population of germ cells that had not entered meiosis, particularly in the periphery of the ovary. It would be interesting to investigate the fate of these peripheral POU5F1+ germ cells at later developmental stages. They may perhaps still enter meiosis or alternatively they may have missed the time window to do so and enter apoptosis. In any case, due to the asynchrony of meiotic entry it is difficult to predict when the expression of the meiotic markers should be expected to occur in the ‘adrenal’ germ cells in humans and we cannot exclude the possibility that ‘adrenal’ germ cells enter meiosis after W22.

### What happens to human ‘adrenal’ germ cells?

Ectopic human KIT+ and POU5F1+ germ cells have been described in the abdomen from W4 to W16 in the developing peripheral nervous system (Mamsen et al., 2012; Mollgard et al., 2010). Here, we show that ectopic germ cells at E15.5 in mice seem to be present in the adrenals, but also at other locations on their migratory path. In humans, although the number of adrenals analyzed was limited, ‘adrenal’ germ cells seemed to be able to downregulate POU5F1 and upregulate DDX4, as in the ovary, but were unable to enter meiosis at least until W21.5. The ‘adrenal’ POU5F1 and/or DDX4 germ cells were not apoptotic (TUNEL-positive). It remains unclear whether during further development, those germ cells remain in the adrenal or lose their germ cell identity by downregulating POU5F1 and DDX4.

Ectopic germ cells are thought to give rise to extragonadal germ cells tumors that occur in particular in midline structures (Looijenga et al., 2013; Schneider et al., 2001), if they fail to be eliminated by apoptosis, but develop instead malignant characteristics. Interestingly, genome-wide association studies (GWAS) have identified single-nucleotide polymorphisms (SNPs) in DDX4 as highly associated with neuroblastoma tumors (Nguyen et al., 2011). Moreover, the origin of adrenal neuroblastoma tumors that occur mainly in children remains elusive, but has been associated with TUBB3-positive neural crest derivatives (De Preter et al., 2006). Because POU5F1+ germ cells both in ovaries and adrenals seem to express TUBB3 as well, it may be important to investigate the expression of additional markers to exclude a potential link between neuroblastoma and ‘adrenal’ germ cells.

## MATERIAL AND METHODS

### Animals and ethics statement

The mouse E15.5 female embryos (*n*=7) used in this study were part of a collaboration with Dr A. McLaren (University of Cambridge, UK, who sadly passed away on 7 July 2007) and were obtained according to the UK Home Office guidelines. The embryos were fixed overnight in 4% paraformaldehyde (PFA, Merck, Darmstadt, Germany) and stored in 100% methanol at −20°C. After rehydration, the embryos were embedded in 4% low-melting agarose (Life Technologies, Eugene, OR, USA) in PBS and were sectioned (200 µm) with a Vibratome HM650V (Microm, Walldorf, Germany). The vibratome sections were boiled 20 min in 0.1 M Tris-HCl, pH 8.5, followed by blocking overnight at 4°C with 0.5% bovine serum albumin (BSA, Life Technologies) diluted in Tris-buffered saline (TBST; 0.14 M NaCl, 2.5 mM KCl, 25 mM Tris pH 7.4) with 0.1% Triton X-100. Then sections were incubated overnight at 4°C with primary antibodies: rabbit anti-DDX4 (1:1000, ab13840, Abcam, Cambridge, UK) and rabbit anti-SYCP3 (1:500, NB300-232, Novus Biologicals Littleton, CO, USA) diluted in 0.5% BSA in TBST. The sections were washed with TBST at 4°C, followed by overnight incubation at 4°C with secondary antibodies: Alexa Fluor 568 goat anti-rabbit IgG (1:500, A-11011, Life Technologies) in 0.5% BSA in TBST, washed with TBST at 4°C and mounted with Vectashield Hard Set Mounting medium with 4′,6-diamidino-2-phenylindole (DAPI) (Vector Laboratories, Burlingame, CA, USA).

### Ethical approval for use of human material

Human fetal gonads (*n*=8 females; *n*=6 males) and adrenal glands (*n*=12 females; *n*=11 males) (Table S1) were donated for research with informed consent from elective abortions without medical indication. The gestational age in weeks and days (for example W8.4 means 8 weeks and 4 days) was determined prior to the procedure by obstetric ultrasonography. To obtain ‘weeks post conception’ one needs to subtract two weeks from the given ‘gestational age’, determined by the last menstrual period (LMP). Both collection and use of human fetal tissues was approved by the Medical Ethical Committee of the Leiden University Medical Center (P08.087).

### Sex genotyping, histology and immunofluorescence

The sex genotyping of the human fetal tissues and processing for histology and immunofluorescence were performed essentially as previous described (Heeren et al., 2015). All immunofluorescence stainings were performed using 0.01 M citric buffer pH 6.0 for antigen retrieval. The primary antibodies used were goat anti-DDX4 (1:1000, AF2030, R&D Systems, Minneapolis, MN, USA), rabbit anti-DDX4 (1:500, ab13840, Abcam, Cambridge, UK), goat anti-POU5F1 (1:100, sc-8628, Santa Cruz Biotechnology, CA, USA), rabbit anti-SYCP3 (1:500, NB300-232, Novus Biologicals Littleton, CO, USA), mouse anti-H2AFX (Ser139) (1:500, 05-636, Millipore Temecula, CA, USA) and mouse anti-TUBB3 (1:500, ab78078, Abcam, Cambridge, UK). The secondary antibodies used were Alexa Fluor 594 donkey anti-goat (1:500, A11058, Life Technologies), Alexa Fluor 488 donkey anti-rabbit (1:500, A21206, Life Technologies), Alexa Fluor 594 donkey anti-mouse (1:500, A-21203, Life Technologies), Alexa Fluor 647 donkey anti-rabbit (1:500, A-31573, Life Technologies), Alexa Fluor 488 donkey anti-goat (1:500, A-11055, Life Technologies), Alexa Fluor 555 donkey anti-rabbit (1:500, A-31572, Life Technologies) and Alexa Fluor 488 donkey anti-mouse (1:500, A21202 Life Technologies). Negative controls were performed by omitting the primary antibodies. TUNEL (terminal deoxynucleotidyl transferase dUTP nick end labeling) assay was performed using In Situ Cell Death Detection Kit (Roche Diagnostics, Mannheim, Germany). The sections were incubated at 37°C for 1 h. All slides were counterstained with a 1:1000 dilution of DAPI (Vector Laboratories, Peterborough, UK) in PBS for 1 min and mounted with ProLongGold antifade reagent (Life Technologies).

### Imaging and quantification

After immunofluorescence, slides were analyzed and photographed on a Leica DMRA fluorescence microscope (Leica, Wetzlar, Germany) with the CoolSnap HQ2 camera (Photometrics, Tucson, USA) or on a Leica AF6000 fluorescence microscope equipped with a Hamamatsu EM-CCD C9100 digital Camera (Leica Microsystems, Wetzlar, Germany). The slides (consecutive for first trimester and every 5-10th for second trimester) were scanned on a Panoramic MIDI digital scanner (3DHISTECH Ltd., Budapest, Hungary) and analyzed with the software program ‘Panoramic viewer’ (3D HISTECH, Budapest, Hungary). Using this software, that allowed digital zooming and channel selection, we quantified the total number of germ cells per scanned section. Examples of POU5F1+DDX4−/low, POU5F1+DDX4+, DDX4+ germ cells are presented in Fig. 4. To be considered a ‘germ cell’, POU5F1 was nuclear, DDX4 was cytoplasmic, DAPI (chromatin condensation) was adequate to the stage of development, both cell and nucleus had the typical germ cell shape and size; the immunostaining pattern was not observed in the negative controls.

### Expression analysis

From online available single cell RNA sequencing data of human gonadal cells of first and second trimester (*n*=328 cells including somatic and germ cells), the expression data in fragments per kilobase of transcript per million (FPKM) of all the female germ cells (*n*=93) was downloaded from the Gene Expression Omnibus (GEO) database (GEO: GSE63818) (Guo et al., 2015). For this analysis all male germ cells (*n*=149) and gonadal somatic cells (*n*=86) were excluded. Genes involved in neural crest cell development, migration and differentiation were obtained based on the GO_BP annotation (GO:0014033, GO:0001755, GO:0014033) from http://www.ensembl.org/biomart. The heatmap showing the log2(FPKM+1) was generated with the R package gplots.
